# Straightforward and immediate ultrasound-guided kidney biopsy using a guide needle technique to get adequate tissue with reduced procedural time

**DOI:** 10.1007/s10157-024-02544-0

**Published:** 2024-08-22

**Authors:** Hiroaki Komatsu, Tomohisa Yamashita, Arata Osanami, Chikako Akazawa, Kota Endo, Shun Tsugawa, Ayumu Kimura, Daisuke Miyamori, Koki Abe, Satoko Takahashi, Yufu Gocho, Masayuki Koyama, Tatsuya Sato, Marenao Tanaka, Norihito Moniwa, Masato Furuhashi

**Affiliations:** 1 Department of Cardiovascular, Renal, and Metabolic Medicine, Sapporo Medical University School of Medicine, South-1, West-16, Chuo-ku, Sapporo, 060-8556 Japan; 2 Department of Nephrology and Dialysis Therapy, Sapporo Central Hospital, Sapporo, Japan; 3 Department of Public Health, Sapporo Medical University School of Medicine, Sapporo, Japan; 4 Department of Cellular Physiology and Signal Transduction, Sapporo Medical University School of Medicine, Sapporo, Japan

**Keywords:** Kidney biopsy, Needle biopsy, Guide needle, Nephrology/education, Ultrasonography

## Abstract

**Background:**

A streamlined and effective renal biopsy technique is essential for all nephrologists, particularly those who are less experienced, such as residents. Herein, we report the efficacy of a Straightforward and Immediate ultrasound-guided kidney biopsy using a Guide Needle (SIGN) technique, which allows operators to insert a biopsy gun through a guide needle placed into the fascia of the posterior abdominal wall.

**Methods:**

A retrospective cross-sectional study was conducted at a nephrology training institution to compare the time spent on the procedure and the number of glomeruli obtained between a group using the SIGN (*n *= 81) and a group using the conventional ultrasound-guided kidney biopsy technique with a needle guide device (*n* = 143).

**Results:**

The median procedure time in the SIGN group (2 min, interquartile range [IQR]: 1–3 min) was significantly shorter than that in the conventional group (3 min, IQR: 2–4 min) (*P* < 0.001). Multivariable linear regression and logistic regression analyses adjusted for covariates, including operators (board-certificated nephrologists or nephrology residents), showed that the use of the SIGN technique was independently associated with a high number of glomeruli obtained and a procedure time above 2 min as the median value (odds ratio: 0.17, 95% confidence interval CI 0.09–0.34). The prevalence of complications was comparable between the two groups (*P* = 0.681).

**Conclusion:**

The SIGN technique reduces the procedure time and obtains adequate biopsy tissue regardless of the operator’s experience. SIGN can be applied in nephrology training programs and used as a standard biopsy technique.

**Supplementary Information:**

The online version contains supplementary material available at 10.1007/s10157-024-02544-0.

## Introduction

Kidney biopsy is an invaluable tool and the gold standard for diagnosing pathological kidney diseases [1–3]. Several modifications have been performed in the procedure of percutaneous kidney biopsy since the landmark publication by Iverson and Brun in 1951 [4]. Pursuing safer and more reliable renal tissue collection has led to the establishment of current ultrasound-guided renal biopsy methods. Furthermore, using a needle guide device that directs needle passage through a predetermined track has enhanced biopsy adequacy and maintained a major complication rate of less than 5% in native kidney biopsies [5–7].

These technical advances have enabled operators to perform kidney biopsies with confidence. However, it has been observed recently that a significant portion of these biopsies are performed by physicians, predominantly radiologists, rather than nephrologists. This trend is noteworthy, given that proficiency in conducting kidney biopsies is a mandatory component of nephrology fellowship training [2]. Furthermore, there has been ongoing debate about whether kidney biopsy should remain a required component of nephrology training programs [8, 9]. Obaidai et al. have argued that nephrologists need to be interested in the progress of procedures and improve their skills to foster patient-provider communication, improve diagnosis, and train the next generation of nephrologists [8]. Under these circumstances, establishing a simple and efficient renal biopsy technique is necessary for all nephrologists, including less experienced residents.

Here, we report a new renal biopsy procedure called Straightforward and Immediate ultrasound-guided renal biopsy using a Guide Needle (SIGN), which allows operators to insert a biopsy gun repeatedly through a guide needle placed in the fascia of the posterior abdominal wall. In the present study, we retrospectively investigated whether the SIGN technique performed by nephrologists, including nephrology residents, can safely shorten the procedure time and obtain more glomeruli than the conventional ultrasound-guided kidney biopsy technique using a needle guide device, as reported by Prasad et al. in 2015 [7].

## Materials and methods

This cross-sectional study conformed to the principles outlined in the Declaration of Helsinki and was approved by the Institutional Ethics Committee of Sapporo Medical University (number 352-139). Written informed consent was obtained from all participants before the procedure. Patients who underwent real-time ultrasound-guided native renal biopsy at our hospital between April 2016 and March 2022 were enrolled (*n* = 256). Participants lacking information and those with no records of procedural details were excluded.

### Preparation for kidney biopsy

**Fig. 1:**
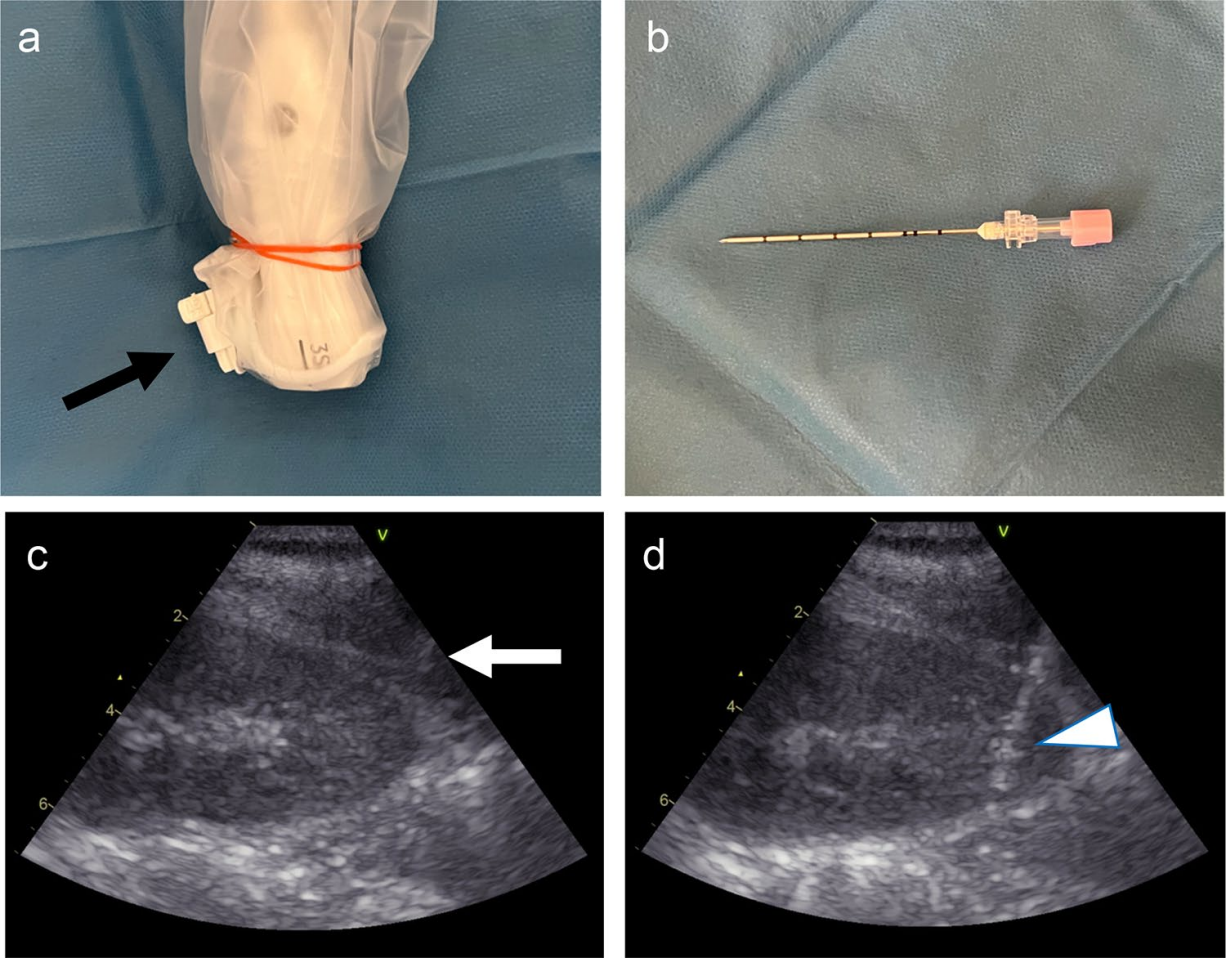
Procedural information of the SIGN technique** a** An image of the Ultra-pro II needle guidance system (black arrow) attached to a sector probe. **b** Guide needle image. **c** Ultrasonography image of the lower pole of the left kidney with a guide needle placed in the fascia of the posterior abdominal wall (white arrow). **d** A Straightforward and Immediate ultrasound-guided kidney biopsy with a Guide Needle (SIGN) technique was performed. The white arrowhead indicates the tip of the biopsy gun

Biopsies were performed by four board-certificated nephrologists from the Japanese Society of Nephrology and four nephrology residents who were supervised by board-certificated nephrologists while performing the procedures. All biopsy procedures were done under real-time ultrasound guidance using Vivid S60 (GE Healthcare, Illinois, USA). An Ultra-pro IITM Needle Guidance System (Civco, Iowa, USA) was attached to a sector probe (3S-RS, GE Healthcare, Illinois, USA) (Fig. 1a). An 18-gauge automated spring-loaded biopsy gun with a stroke length of 22 mm (BARD MaxCore, cat# MC1820, Bard Peripheral Vascular Inc., Bethesda, MD, USA) was used. When the kidney appeared atrophic, another gun with a stroke length of 11 mm (BARD Monopty, cat# 211,820; Bard Peripheral Vascular Inc.) was used.

All patients were admitted for at least one day before the biopsy. Antiplatelet or anticoagulant drugs were adequately withdrawn [3]. When the platelet count was less than 50,000/µL, the biopsy procedure was performed after platelet transfusion [10]. The patients were placed in the prone position; the puncture site was disinfected with 10% povidone-iodine, and local anesthesia with 1% lidocaine was administered. When systolic blood pressure was higher than 140 mmHg prior to the biopsy, continuous intravenous nicardipine was administered with a target of 140 mmHg or less [11].

### Kidney biopsy procedures

The operators independently determined the use of the conventional technique or SIGN technique. The conventional technique was performed as previously described [7]. In summary, following the administration of local anesthesia and a minor skin incision, a biopsy gun was inserted to obtain a biopsy core from the lower pole of the kidney. This process involved utilizing a needle guide attached to the ultrasound probe held by an assistant. As the assistant applied pressure to the puncture site to control bleeding, the collected specimen was carefully deposited onto sterile gauze. This procedure was then repeated.

The typical procedure for the SIGN technique is shown in a video (Supplemental Material 1). After administering local anesthesia, a 17-gauge guide needle (TSK Laboratory, Tochigi, Japan) was inserted through the needle guide without making a skin incision, while holding an ultrasound probe with a needle guide device in the non-dominant hand (Fig. 1b). A guide needle was then placed in the fascia of the posterior abdominal wall (Fig. 1c). A biopsy gun was inserted through the guide needle into the lower pole of the kidney to obtain the biopsy core (Fig. 1d). The biopsy gun was extracted and subsequently handed over to the assistant. By securing the probe to the patient's back through a guide needle, the operator could maintain focus on the ultrasound monitor, facilitating the prompt execution of successive punctures immediately after the assistant returned the biopsy needle. The versatility of this technique allows for puncturing various sites within the kidney by adjusting the probe's tilt and modifying the breathing depth.

After renal biopsy, the puncture site was pressed down for 5 min, and a flat dressing was applied. The patient was placed in the supine position. Blood tests were performed 3 h after the procedure. The following day, an ultrasound examination was conducted to detect any signs of major hematoma.

### Outcomes

The procedure time was calculated by subtracting the time of the first puncture from that of the last puncture and recorded in minutes. The time required for administering local anesthesia was not included in the procedure. The number of glomeruli obtained from the light and fluorescence microscopy specimens was counted by kidney pathology specialists who did not have any procedural information. Major complications were defined as the requirement for unplanned blood product transfusion or invasive procedures by radiographic or surgical intervention within 24 h after the biopsy, acute renal obstruction, extended hospitalization, septicemia, or death. Minor complications were defined as transient gross hematuria, a decrease in hemoglobin concentration of ≥ 2.0 g/dL, or vagal reflex [12]. When a patient encountered more than one complication, each specific complication was considered and accounted for separately.

### Measurements

Patient information, including age, sex, body mass index (BMI), medical history, blood pressure before the procedure, and blood test results were obtained from electronic medical charts. The estimated glomerular filtration rate (eGFR) was calculated from serum creatinine levels using an equation for Japanese [13]. Procedural details regarding operators, procedure time, number of punctures, and number of complications were also collected from electronic medical charts. Two nephrologists independently measured and averaged the kidney depth and volume in computed tomography images. Kidney depth was defined as the average perpendicular distance from the dorsal skin to the most anterior and posterior points of the kidney at the level of the renal hilum [14]. Kidney volume was calculated by the following formula: *π*/6 × length × width × depth [15]. To evaluate the influence of operator experience on outcomes, we employed the distinction between board-certificated nephrologists affiliated with the Japanese Society of Nephrology and nephrology residents. This categorization served as an independent variable in our analysis. Table S1 presents the number of kidney biopsies and years of nephrology residency experience at the time of the first biopsy for each nephrology resident during the study period. High-risk factors for bleeding complications were defined as (1) pre-biopsy blood pressure over 140 mmHg, (2) the presence of diabetes mellitus [16], and (3) a composite of high-risk conditions known to increase bleeding risk as previously reported [3], including malignant hypertension, anti-neutrophil cytoplasmic antibody-associated vasculitis, lupus nephritis with vasculitis, amyloidosis, multiple myeloma, thrombotic microangiopathy, polycythemia vera, antiphospholipid syndrome, liver cirrhosis, thrombocytopenia, anasarca, myelofibrosis, renal dysfunction, and organomegaly syndrome.

### Statistical analyses

The Shapiro–Wilk test was used to determine whether a continuous variable followed a normal distribution. Values with and without normal distribution are expressed as means ± standard deviation and median (interquartile range [IQR]), respectively. In univariate analyses between the two groups, continuous variables with normal and non-normal distribution were examined using the Student's t-test and Mann–Whitney *U* test, respectively. In the univariate analyses among several groups, continuous variables with a normal distribution and those with a non-normal distribution were examined using one-way analysis of variance with post-hoc Holm's method and the Kruskal–Wallis test with Steel–Dwass’s method, respectively. Categorical variables were examined using Fisher's exact test. Multiple linear and logistic regression analyses were performed to determine the independent factors of the outcomes. All data were analyzed by the EZR [17]. A *p*-value of less than 0.05 was considered statistically significant.

## Results

### Characteristics of the study patients

**Fig. 2:**
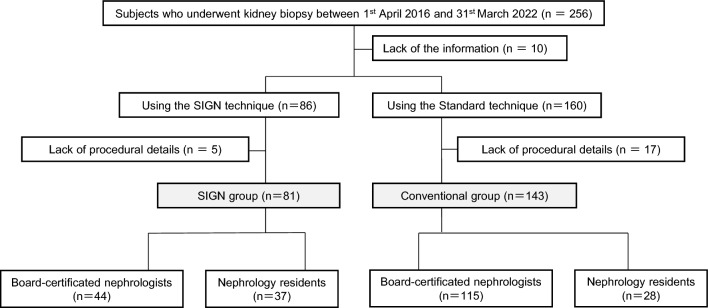
Flow chart of study subjects Among 256 individuals who underwent kidney biopsy between April 1, 2016, and March 31, 2022, 224 patients were finally recruited for analyses in the present study

A flowchart of the study participants is shown in Fig. 2. After excluding individuals lacking information or procedural details, 81 and 143 patients were assigned to the SIGN and conventional groups, respectively. The baseline characteristics of the recruited patients are presented in Table 1. The mean age of the SIGN group was lower than that of the conventional group (*P* = 0.009). The prevalence of kidney depth above the median tended to be higher in the SIGN group than in the conventional group, but the difference was not statistically significant (*P* = 0.051). There were no significant differences in sex, BMI, clinical diagnosis, kidney volume, eGFR, hemoglobin level, or high-risk factors for bleeding complications. There was no significant difference in the number of punctures between the SIGN (median: 4 [IQR: 4–5]) and conventional groups (median: 4 [IQR: 4–5]) (*P* = 0.725) (Table 1).

**Table 1:** Baseline clinical and laboratory characteristics of the subjects

	SIGN group (*n* = 81)	Conventional group (*n* = 143)	*P*
Age (years)	58 [38, 70]	64 [47, 74]	0.009
Age ≥ 75	9 (11.0)	35 (24.4)	0.022
Male	41 (50.6)	74 (51.7)	0.890
BMI (kg/m^2^)	23.8 ± 4.4	23.4 ± 4.1	0.483
BMI ≥ 25	28 (34.1)	50 (35)	0.764
Clinical diagnosis			0.756
Chronic glomerulonephritis	46 (49.7)	71 (56.8)	0.332
Nephrotic syndrome	16 (24.5)	35 (19.8)	0.508
RPGN/AKI	12 (17.5)	25 (14.8)	0.709
Others	7 (8.4)	12 (8.6)	1
Kidney depth (mm)	76 ± 16	73 ± 17	0.144
Kidney depth above the median	48 (59.2)	64 (44.8)	0.051
Kidney volume (cc)	155 [124, 194]	142 [122, 177]	0.078
Biochemical Data			
eGFR (ml/min/1.73 m^2^)	46.6 [29.9, 77.0]	42.3 [21.2, 67.0]	0.139
eGFR < 30	21 (25.9)	54 (37.8)	0.078
Hemoglobin (g/dL)	11.9 ± 2.1	11.4 ± 2.2	0.078
High-risk factors for bleeding complications			
Pre-biopsy sBP ≥ 140 mmHg	34 (41.9)	49 (34.3)	0.254
Diabetes mellitus	14 (17.2)	23 (16.1)	0.852
High-risk conditions^a^	13 (16.0)	28 (19.5)	0.591
Board-certificated nephrologists	44 (54.3)	115 (80.4)	< 0.001
Nephrology residents	37 (45.7)	28 (19.6)	< 0.001
Number of punctures	4 [4, 5]	4 [4, 5]	0.725

Variables are expressed as numbers (%), means ± SD, or medians [interquartile range]

SIGN, The Straightforward and Immediate ultrasound-guided kidney biopsy using a Guide Needle technique; *BMI* body mass index, *RPGN* rapidly progressive glomerulonephritis, *AKI* acute kidney injury, *eGFR* estimated glomerular filtration rate, *sBP* systolic blood pressure

^a^The composites of high-risk conditions for bleeding complications, including malignant hypertension, anti-neutrophil cytoplasmic antibody (ANCA)-associated vasculitis, lupus nephritis with vasculitis, amyloidosis, multiple myeloma, thrombotic microangiopathy, polycythemia vera, antiphospholipid syndrome, and liver cirrhosis, and thrombocytopenia, anasarca, myelofibrosis, renal dysfunction, and organomegaly (TAFRO) syndrome

Among the nephrology residents, the number of subjects for whom the SIGN technique was selected was significantly higher than that of the conventional technique (56.9% vs. 43.1%) (*P* < 0.001) (Supplementary Table S2). Conversely, the number of board-certificated nephrologists who selected the SIGN technique was significantly lower than those who selected the conventional technique (27.7% vs. 72.3%) (*P* < 0.001) (Supplementary Table S2).

The baseline characteristics of the recruited participants, divided by board-certificated nephrologists and nephrology residents, are shown in Table 2. The patients in the SIGN group of nephology residents were significantly younger and had a considerably higher eGFR than those in the conventional group of board-certificated nephrologists (Table 2).

**Table 2:** Baseline characteristics among the four groups

	Board-certificated nephrologist (*n *= 159)		Nephrology residents (*n* = 65)		P*
	SIGN group (*n* = 44)	Conventional Group (*n *= 115)	SIGN group (*n* = 37)	Conventional group (*n* = 28)	
Age (years)	63 [40, 72]	65 [49, 76]	47 [38, 64] **	64 [46, 72]	0.013
Age ≥ 75	6 (13.6)	32 (27.8)	3 (8.1)	3 (10.7)	0.019
Male	24 (54.5)	60 (52.2)	17 (45.9)	14 (50)	0.887
BMI (kg/m^2^)	23.8 ± 4.3	23.6 ± 4.2	23.7 ± 4.5	22.1 ± 3.4	0.338
BMI ≥ 25	14 (31.8)	42 (36.5)	13 (35.1)	8 (28.6)	0.868
Clinical diagnosis					
Chronic glomerulonephritis	23 (52.3)	58 (50.4)	23 (62.2)	13 (46.4)	0.581
Nephrotic syndrome	10 (22.7)	29 (25.2)	6 (16.2)	6 (21.4)	0.760
RPGN/AKI	7 (15.9)	20 (17.4)	5 (13.5)	5 (17.9)	0.964
Others	4 (9.1)	8 (7.0)	3 (8.1)	4 (14.3)	0.619
Kidney depth (mm)	77 ± 16	74 ± 17	76 ± 15	69 ± 14	0.255
Kidney depth above the median	27 (61.4)	55 (47.8)	21 (56.7)	9 (32.1)	0.081
Kidney volume (mm^2^)	145 [123, 195]	142 [125, 180]	163 [130, 193]	150 [112, 174]	0.722
Biochemical Data					
eGFR (ml/min/1.73 m^2^)	35 [26, 62]	41 [21, 64]	61 [33, 79]**	54 [35, 79]	0.007
eGFR < 30	16 (36.4)	48 (42.7)	5 (13.7)**	6 (21.4)	0.005
Hemoglobin (g/dL)	11.7 ± 2.0	11.2 ± 2.3	12.1 ± 2.1	11.7 ± 1.6	0.201
Pre-biopsy sBP ≥ 140 mmHg	18 (40.9)	41 (35.7)	16 (43.2)	8 (28.6)	0.606
Diabetes mellitus	10 (22.7)	19 (16.5)	4 (10.8)	4 (14.3)	0.566
High-risk conditions^a^	5 (11.4)	21 (18.2)	8 (21.2)	7 (25.0)	0.440
Number of punctures	4 [4, 5]	4 [4, 5]	4 [4, 5]	4 [4, 5]	0.050

Variables are expressed as numbers (%), means ± SD, or medians [interquartile range]

SIGN group, The Straightforward and Immediate ultrasound-guided kidney biopsy using a Guide Needle technique group; BMI, body mass index; RPGN, rapidly progressive glomerulonephritis; AKI, acute kidney injury; eGFR, estimated glomerular filtration rate; sBP, systolic blood pressure

^*^P values were calculated by one-way analysis of variance test or Kruskal–Wallis’s test among four groups

**P < 0.05 vs conventional technique by board-certificated nephrologists

^a^The composites of high-risk conditions for bleeding complications including malignant hypertension, anti-neutrophil cytoplasmic antibody (ANCA)-associated vasculitis, lupus nephritis with vasculitis, amyloidosis, multiple myeloma, thrombotic microangiopathy, polycythemia vera, antiphospholipid syndrome, liver cirrhosis, and thrombocytopenia, anasarca, myelofibrosis, renal dysfunction, and organomegaly (TAFRO) syndrome

### Complications

The prevalence of major complications in the SIGN group was 1.2%, comparable to that in the conventional group (2.1%). No significant differences in other complications were found between the two groups (Table 3). No significant differences were observed in complications among the four groups divided according to the use of the SIGN method and whether the operator was a board-certificated nephrologist or nephrology resident (Supplementary Table S3).

**Table 3:** Complications

	SIGN group (*n* = 81)	Conventional group (*n* = 143)	*P*
Total complications	9 (11.1)	19 (13.3)	0.681
Major complications	1 (1.2)	3 (2.1)	1
Acute renal obstruction	1 (1.2)	0 (0)	0.862
Unplanned blood transfusions within 24 h after the biopsy	0 (0)	3 (2.1)	0.556
Extended hospitalization	0 (0)	0 (0)	1
Minor complications			
Gross hematuria	5 (6.1)	11 (7.6)	0.791
Decrease in hemoglobin level of 2.0 g/dL or greater	1 (1.2)	3 (2.1)	1
Vagal reflex	3 (3.7)	4 (2.8)	0.705

Variables are expressed as numbers (%)

SIGN group, The Straightforward and Immediate ultrasound-guided kidney biopsy using a Guide Needle technique group

### Comparison of the procedure time between the SIGN and conventional groups

**Fig. 3:**
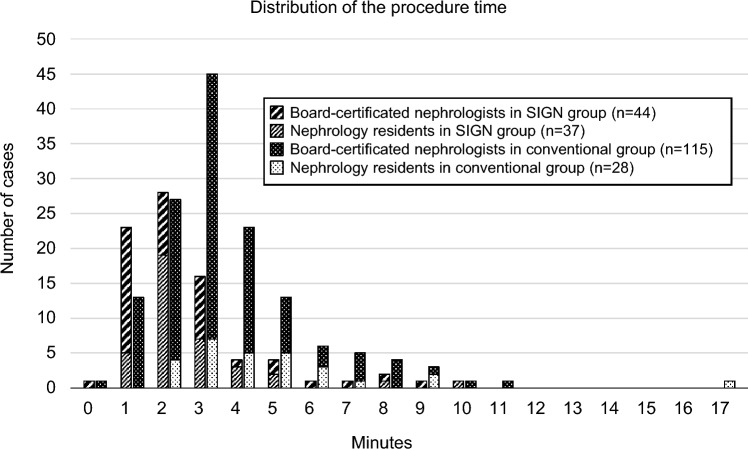
Distribution of the procedure time Distribution of the procedure time in all patients who underwent kidney biopsy using a conventional technique or the Straightforward and Immediate ultrasound-guided kidney biopsy using a Guide Needle (SIGN) technique by both board-certificated nephrologists and nephrology residents

The distribution of procedure time is shown in Fig. 3. The procedure time was significantly shorter in the SIGN group (median: 2 min [IQR: 1–3 min]) than in the conventional group (median: 3 min [IQR: 2–4 min], *P* < 0.001). The incidence of procedure times exceeding the median duration of 2 min among all patients was notably lower in the SIGN group compared to the conventional group (37.0% vs. 71.3%, *P* < 0.001).

**Fig. 4:**
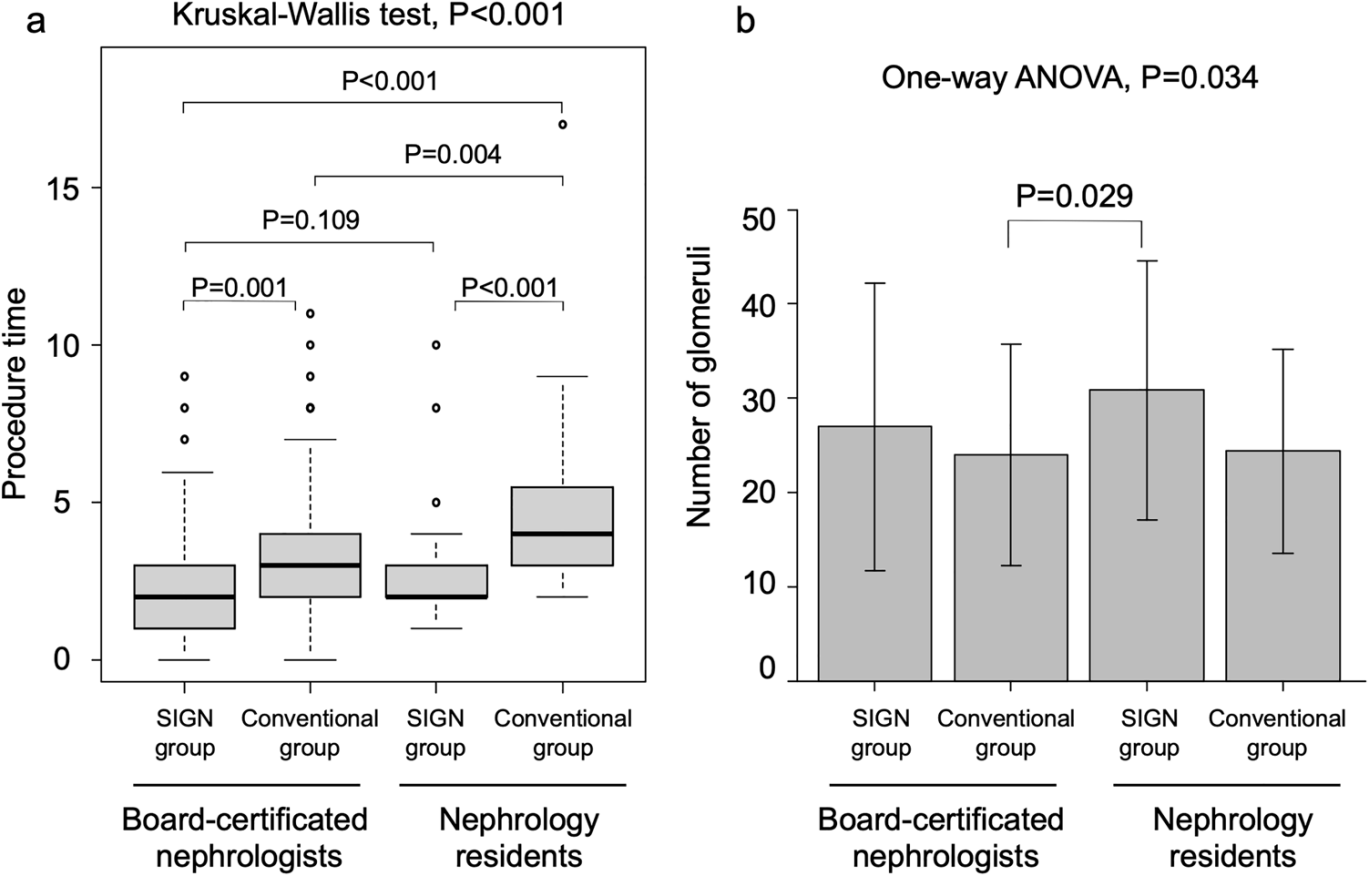
Comparisons of the procedure time and the number obtained glomeruli** a** Comparison of the procedure time in patients who underwent kidney biopsy using a conventional technique or the Straightforward and Immediate ultrasound-guided kidney biopsy using a Guide Needle (SIGN) technique in both board-certificated nephrologists and nephrology residents. **b** Comparison of the number of glomeruli obtained using a conventional or SIGN technique in both board-certificated nephrologists and nephrology residents

The SIGN technique significantly shortened the procedure time for board-certificated nephrologists and residents. When using the conventional method, the procedure time was considerably longer in nephrology residents compared to board-certificated nephrologists (*P* = 0.004). Conversely, no statistical differences were observed between board-certificated nephrologists and residents when employing the SIGN method (*P* = 0.109) (Fig. 4a).

### Comparison of the number of glomeruli between the SIGN and conventional groups

The number of glomeruli obtained was significantly greater in the SIGN group (29 ± 15) than in the conventional group (24 ± 12) (*P* = 0.008). The number of glomeruli divided by the number of punctures was significantly higher in the SIGN group (7 ± 4) than in the conventional group (6 ± 3) (*P* = 0.027) (Table 4). The glomeruli obtained were significantly higher in the SIGN group of nephrology residents than in the conventional group of board-certificated nephrologists (Fig. 4b).

**Table 4:** The number of glomeruli between the SIGN and conventional groups

	SIGN group (*n* = 81)	Conventional group (*n* = 143)	*P*
The number of glomeruli	29 ± 15	24 ± 12	0.008
The number of glomeruli divided by the number of punctures	7 ± 4	6 ± 3	0.027

Variables are expressed as numbers, means ± SD

SIGN group, The Straightforward and Immediate ultrasound-guided kidney biopsy using a Guide Needle technique group

### Determinants for the procedure time and the number of glomeruli

Logistic regression analysis, adjusting for age, sex, BMI, kidney depth, kidney volume, operator experience, and eGFR showed that the use of the SIGN technique was independently associated with a significantly reduced likelihood of procedure times exceeding 2 min (odds ratio 0.17, 95% confidence interval CI 0.09–0.34) (Table 5).

**Table 5:** Logistic regression analysis for the puncture time above 2 min

	Odds ratio	95%CI	*P*
SIGN technique	0.17	0.09–0.34	< 0.001
Board-certificated nephrologists	0.59	0.29–1.21	0.151
Age ≥ 75	0.51	0.24–1.10	0.085
Male	1.66	0.89–3.07	0.109
BMI ≥ 25	1.50	0.71–3.16	0.289
Kidney depth above the median	1.01	0.48–2.10	0.989
Kidney volume below the median	0.72	0.39–1.33	0.300
eGFR < 30 (ml/min/1.73 m^2^)	1.48	0.76–2.84	0.244

*CI* confidence interval; Other abbreviations are summarized in Table 1

In multivariable linear regression analysis, adjusting for age, sex, BMI, number of punctures, use of an 11 mm stroke biopsy gun, kidney depth, kidney volume, operator experience, and eGFR showed that the use of the SIGN technique was independently associated with a significantly higher number of glomeruli compared to the conventional technique (estimate: 4.75, 95% CI 0.99–8.50) (Table 6).

**Table 6:** Multiple linear regression for the number of glomeruli

	Estimate	95%CI	*P*
SIGN technique	4.75	0.99–8.50	0.013
Board-certificated nephrologists	− 0.89	− 4.82–3.03	0.653
Age (per 1 year-old)	− 0.06	− 0.17–0.05	0.279
Male	− 1.07	− 4.54–2.40	0.544
BMI ≥ 25	− 3.06	− 7.32–1.18	0.156
Number of punctures	0.66	− 1.40–2.73	0.526
The use of an 11 mm stroke biopsy gun	− 7.88	− 17.8–2.02	0.118
Kidney depth above the median	− 1.99	− 6.14–2.15	0.343
Kidney volume below the median	0.53	− 2.93–3.99	0.762
eGFR (ml/min/1.73 m^2^)	0.03	− 0.03–0.10	0.294

*CI* confidence interval; Other abbreviations are summarized in Table 1

## Discussion

The present study showed that the SIGN technique, a real-time ultrasound-guided renal biopsy method described here for the first time, reduced the procedure time and increased the yield of glomeruli without an increase in complication rates. Importantly, nephrologists, including residents with limited experience, performed the biopsies, not radiologists, and the operators voluntarily opted for this technique in our study.

The reduction in the procedure time when using the SIGN technique may be due to three reasons. First, operators could perform the procedure without resistance when penetrating the fascia with the biopsy gun because the guide needle had already penetrated the fascia. Second, the probe was secured to the patient's back using a guide needle, eliminating the need to relocate the kidneys. Third, the operators could keep their eyes on the monitor. The reasons why kidney biopsy using the SIGN method obtained more glomeruli than the conventional method may include two key factors. First, by securing the probe alongside the guide needle, the operator maintains a clear and stable renal visualization, as illustrated in Supplemental Material 1. This enables precise placement of the biopsy needle at the optimal site, potentially yielding sufficient tissue samples from the kidney. Second, the short procedure time allowed operators to complete the procedure before uncomplicated hematoma formation, a common occurrence in many cases [18–21]. This could be another reason for the significantly higher number of glomeruli obtained in the SIGN group, as even a small hematoma made it difficult for the operator to puncture the appropriate site. As demonstrated in the video describing the SIGN technique (Supplemental Material 1), this method consistently provides clear ultrasound images of the kidney until the final puncture. We hypothesize that the brief procedure duration enables the completion of all punctures before the hematoma formation which could otherwise hinder the procedure.

Furthermore, the SIGN group tended to include more patients with kidney depths above the median, indicating a likelihood of requiring deeper punctures. Despite encountering more challenging cases in terms of kidney depth, the SIGN technique’s shorter procedure time compared to the conventional method and the sufficient number of glomeruli obtained suggest its effectiveness in patients necessitating deeper punctures.

For adequate evaluation, at least 8–10 glomeruli are required in patients with IgA nephropathy [22], lupus nephritis [23], and diabetic nephropathy [24]. Furthermore, in patients with focal lesions, at least 25 glomeruli are required for an accurate diagnosis [25]. Therefore, a high number of glomeruli are essential for accurate pathological diagnosis. Interestingly, nephrology residents performed the SIGN technique more frequently than board-certificated nephrologists (Supplementary Table S2), possibly due to the above reasons. Thus, the SIGN technique can be used in nephrology training programs.

It has been reported that nephrologists performed 91% of renal biopsies in the 1990s [26]. However, the role of kidney biopsy in nephrology training has been discussed in recent years [2, 8, 9]. In this study, board-certificated nephrologists tended to use conventional techniques (Supplementary Table S2). One possible reason for this is the indifference to the procedure's progress. Procedures that have been performed for many years continue without modifications. In the present study, nephrology residents preferred the SIGN technique, and the number of glomeruli obtained by nephrology residents using the SIGN technique was more significant than that obtained by board-certificated nephrologists using the conventional technique. The SIGN technique has the potential to become the next-generation standard.

The SIGN technique was inspired by the coaxial technique described by a radiologist in 2008 for kidney and liver biopsies, in which the physician inserts a guide needle directly into the target organ [27]. Babaei et al. reported that the coaxial technique reduced the complication rate and procedural time compared to the non-coaxial technique in percutaneous renal biopsy [28]. A direct comparison could not be made because they did not define the procedure time. However, the median procedure time of 2 min in the present study was shorter than the mean procedure time of 5 min reported by Babaei et al. [28]. In addition, the original coaxial technique may misdiagnose focal diseases because it obtains tissues from precisely one location in the kidneys. This problem was solved using the SIGN technique because specimens can be obtained from different kidney sites by tilting the probe. Since the present study did not aim to compare the coaxial technique with the SIGN technique, further studies are needed to determine whether intra-renal or extra-renal placement of the guide needle is superior.

This study had several limitations. First, the choice of procedure technique was based on operator preference, potentially introducing bias in procedure time and number of glomeruli obtained. However, blinding the assessment of procedure time was impractical. A strength of this study was the inclusion of multiple physicians with varying experience levels, including nephrology residents who were performing their first renal biopsy. Second, procedure time was not recorded in seconds, limiting a more precise evaluation. Third, there may have been selection bias as patients in the SIGN group treated by nephrology residents were younger and had higher eGFR compared to those in the conventional group. Nonetheless, we mitigated this potential bias by conducting multivariate analysis adjusting for covariates such as age and eGFR to assess the procedure time and number of glomeruli obtained.

In conclusion, using the SIGN technique in renal biopsy can shorten the procedure time and increase the number of glomeruli obtained, with no increase in complication rates compared with conventional techniques. Therefore, the SIGN technique can be used in nephrology training programs. The preference of nephrology residents for SIGN, a modified coaxial technique, indicates the ease of the procedure and encourages future nephrologists.

## Supplementary Information

Below is the link to the electronic supplementary material.

Supplementary file1 (MP4 189251 KB)

Supplementary file2 (DOCX 20 KB)
