# Supplementary material for: Straightforward and immediate ultrasound-guided kidney biopsy using a guide needle technique to get adequate tissue with reduced procedural time
Source: Clin Exp Nephrol. 2024 Aug 22;29(1):57–66. doi: 10.1007/s10157-024-02544-0 (PMC11807059; doi:10.1007/s10157-024-02544-0)
Supplement: Supplementary file 2 — Supplementary file2 (DOCX 20 KB) [file 10157_2024_2544_MOESM2_ESM.docx]

| Supplementary Table S1. Information about nephrology residents. | | | | |
| --- | --- | --- | --- | --- |
|  | Nephrology Residents (n = 4) | | | |
|  | Resident 1 | Resident 2 | Resident 3 | Resident 4 |
| Experience as a nephrology resident*　(year) | 0 | 2 | 1 | 1 |
| Number of kidney biopsies during the study period | 46 | 11 | 6 | 2 |
| * Years of experience since admission to the Japanese Society of Nephrology at the time of the first kidney biopsy performed by each resident during the study period. | | | | |

| Supplementary Table S2. Number of subjects for whom the SIGN technique or the conventional technique was selected in board-certificated nephrologists and nephrology residents. | | | |
| --- | --- | --- | --- |
|  | Board-certificated Nephrologists  (n = 159) | Nephrology Residents　　　　 (n = 65) | P |
| SIGN group | 44 (27.7) | 37 (56.9) | <0.001 |
| Conventional group | 115 (72.3) | 28 (43.1) | <0.001 |
| Variables are expressed as numbers (%). | | | |
| Abbreviations: SIGN group, Straightforward and Immediate ultrasound-guided kidney biopsy using a Guide Needle technique group. | | | |

| Supplementary Table S3. Complications among the four groups. | | | | | | |
| --- | --- | --- | --- | --- | --- | --- |
|  | Board-certificated Nephrologists (n = 159) | |  | Nephrology Residents (n = 65) | |  |
|  | SIGN group (n = 44) | Conventional group (n = 115) |  | SIGN group  (n = 37) | Conventional group (n = 28) | P |
| Total complications | 4 (9.1) | 18 (15.7) |  | 5 (13.5) | 1 (3.5) | 0.312 |
| Major complications | 0 (0) | 3 (2.6) |  | 1 (2.7) | 0 (0) | 0.589 |
| Acute renal obstruction | 1 (2.3) | 0 (0) |  | 0 (0) | 0 (0) | 0.252 |
| Unplanned blood transfusions within 24 hours after the biopsy | 0 (0) | 3 (2.6) |  | 0 (0) | 0 (0) | 0.412 |
| Extended hospitalization | 0 (0) | 0 (0) |  | 0 (0) | 0 (0) | 1 |
| Minor complications | 4 (9.1) | 15 (13.0) |  | 4 (10.8) | 1 (3.5) | 0.521 |
| Gross hematuria | 3 (6.8) | 10 (8.7) |  | 2 (5.4) | 1 (3.5) | 0.771 |
| Decrease in hemoglobin level of 2.0 g/dL or greater | 0 (0) | 3 (2.6) |  | 1 (2.7) | 0 (0) | 0.589 |
| Vagal reflex | 1 (2.3) | 4 (3.5) |  | 2 (5.4) | 0 (0) | 0.640 |
| P values were calculated by the Kruskal-Wallis’s test among the four groups. | | | | | | |
| Variables are expressed as numbers (%). | | | | | | |
| Abbreviations: SIGN group, Straightforward and Immediate ultrasound-guided kidney biopsy using a Guide Needle technique group. | | | | | | |
